# Austrian tricentric real-life analysis of molecular profiles of metastatic biliary tract cancer patients

**DOI:** 10.3389/fonc.2023.1143825

**Published:** 2023-05-10

**Authors:** Hossein Taghizadeh, Theresa Schmalfuss, Agnieszka Maj-Hes, Josef Singer, Gerald W. Prager

**Affiliations:** ^1^ Division of Oncology, Department of Internal Medicine I, University Hospital St. Pölten, St. Pölten, Austria; ^2^ Karl Landsteiner University of Health Sciences, Krems, Austria; ^3^ Karl Landsteiner Institute for Oncology and Nephrology, St. Pölten, Austria; ^4^ Center for Cancer Research, Medical University of Vienna, Vienna, Austria; ^5^ Comprehensive Cancer Center Vienna, Medical University of Vienna, Vienna, Austria; ^6^ Medical University Vienna, Department of Medicine I, Division of Oncology, Vienna, Austria; ^7^ Department of Pulmonology, Klinik Penzing, Vienna, Austria; ^8^ Division of Oncology, Department of Internal Medicine II, University Hospital Krems, Krems, Austria

**Keywords:** biliary tract cancer, molecular aberrations, molecular profiling, precision medicine, precision oncology

## Abstract

**Introduction:**

Metastatic biliary tract cancer (BTC) is a rare and aggressive entity associated with poor prognosis. It represents a major challenge for adequate treatment strategies. In recent years, BTC has become a model for precision medicine in gastrointestinal oncology. Therefore, the analysis of the individual molecular profile in BTC patients may lead to targeted therapies for the benefit of patients.

**Methods:**

In this Austrian, tricentric, real-world, retrospective analysis, we investigated patients diagnosed with metastatic BTC who underwent molecular profiling between 2013 and 2022.

**Results:**

In total, 92 patients were identified in this tricentric analysis and 205 molecular aberrations, including 198 mutations affecting 89 different genes in 61 patients were found. The predominant mutations were in *KRAS* (n=17; 22.4%), *TP53* (n=17; 22.4%), *PIK3CA* (n=7; 9.2%), *FGFR2* (n=7; 9.2%), *DNMT3A* (n=7; 9.2%), *IDH1* (n=7; 9.2%), *IDH2* (n=6; 7.9%), *CDKN2A* (n=6; 7.9%), *BAP1* (n=4; 5.3%), *NF1* (n=4; 5.3%), and *NF2* (n=4; 5.3%). Three patients had *HER2* amplification. MSI-H status and *FGFR2* fusion genes were each observed in two different patients. One patient had a *BRAF V600E* mutation. Eventually, 10 patients received targeted therapy, of whom one-half derived clinical benefit.

**Conclusions:**

Molecular profiling of BTC patients is implementable in routine clinical practice and should be regularly employed to detect and exploit molecular vulnerabilities.

## Introduction

Biliary tract cancer (BTC) is a highly malignant and fatal cancer that arises from the biliary epithelium in the bile duct, gallbladder, or ampulla of Vater. BTC is a molecularly heterogeneous entity encompassing several subentities: gallbladder carcinoma, distal cholangiocarcinoma (CCA), perihilar CCA, intrahepatic CCA (iCCA), and ampullary carcinoma (1, 2). Each of these subtypes has a distinct molecular signature, highlighting the high spatial heterogeneity of this disease group (3, 4). BTC is a relatively rare cancer, with an incidence of about 2/100,000 in the Western world (5); however, its incidence is clearly increasing. Ouyang et al. investigated the burden of BTC in 195 countries between 1990 and 2017 and reported that the incidence of BTC increased by 76%, mortality increased by 65%, and disability-adjusted life-years increased by 52% from 1990 to 2017 (6). BTC is an aggressive malignancy that causes non-specific symptoms. Therefore, this entity is often diagnosed in the advanced stages. Due to late manifestation of symptomatology, paucity of effective treatments, molecular diversity, and poor understanding of its complex molecular mechanisms and pathways, advanced BTC has a dismal prognosis, with a 5-year survival rate of only 2% (2, 4, 7–9).

For many years, systemic chemotherapy was the mainstay of BTC treatment. Recently, the clinical TOPAZ-1 phase 3 trial demonstrated a mild but statistically significant improvement by the addition of durvalumab to cisplatin plus gemcitabine in first line setting, independent of the primary tumor location. The immunochemotherapy led to significantly increased mPFS (7.2 versus 5.7 months, HR 0.75 [0.63–0.89]) and mOS (12.9 versus 11.3 months, HR 0.76 [0.64–0.91]) and higher OS rate at 24 months, with an improvement of 12.1% (23.6% *vs* 11.5% estimated OS at 24 months) (10). For second line treatment, Lamarca et al. introduced the FOLFOX regime (consisting of folinic acid, 5-fluorouracil, and oxaliplatin), which was evaluated in the ABC-06 phase 3 trial (11).

In recent years, the emergence of next-generation sequencing (NGS), consecutive identification of molecular aberrations, and the development of molecular-guided targeted therapies has heralded the era of precision oncology or molecular oncology.

BTC, particularly small duct iCCA, has evolved as a model disease for molecular oncology, as this entity harbors numerous druggable molecular aberrations. Currently, the targeted drug ivosidenib against *IDH1* mutation targeted drugs is approved by both the Food and Drug Administration (FDA). Pemigatinib directed against *FGFR2* fusions and rearrangements and pembrolizumab indicated for tumors with microsatellite instability-high (MSI-H) or deficient mismatch repair are approved by both FDA and European Medicines Agency (EMA) (12, 13). Other targetable molecular lesions include *BRAF V600E*, *HER2* positivity, and *NTRK* fusions (14). Therefore, molecular profiling is essential for modern-day therapeutic management of BTC, especially after failure of first line treatment, and it is recommended by both ESMO and the and the American Society of Clinical Oncology (ASCO).

In this real-world study, we sought to map the molecular profiles of metastatic BTC cases and to specifically target the detected molecular alterations.

## Materials and methods

### Patients and design of the precision medicine platform

We conducted retrospective analysis of 92 patients with metastatic BTC who underwent molecular profiling at three main Austrian centers: the Medical University of Vienna, the University Hospital St. Poelten, and the University Hospital Krems. Patients needed to have an Eastern Cooperative Oncology Group (ECOG) performance status of 0 or 1. Furthermore, the Institutional Ethics Committee of both Austrian centers approved this analysis (Number 1099/2021). 1

### Cancer gene panel sequencing

DNA was isolated from paraffin-embedded tissue samples using a QIAamp Tissue Kit™ (Qiagen, Hilden, Germany). From each specimen, 10 ng DNA was used for sequencing. In selected cases for which tissue samples were unavailable, liquid biopsy was performed. The genetic profile was generated via Ion AmpliSeq Cancer Hotspot Panel v2 (Thermo Fisher Scientific, Waltham, MA, USA), Ion AmpliSeq™ Cancer Hotspot Panel v3 (Thermo Fisher Scientific, Waltham, MA, USA), Oncomine™ Comprehensive Assay v3 (Thermo Fisher Scientific, Waltham, MA, USA) NGS panel, TruSight Oncology 500 Assay (Illumina, San Diego, CA, USA), Oncomine™ Precision Assay GX (Thermo Fisher Scientific, Waltham, MA, USA), FoundationOne CDx (Foundation Medicine, Cambridge, MA, USA), and FoundationOne Liquid CDx (Foundation Medicine, Cambridge, MA, USA).

The FoundationOne Liquid CDx assay was performed using circulating cell- free DNA (cfDNA) isolated from plasma derived from anti-coagulated peripheral whole blood from patients with solid malignant neoplasms. The assay employed a single DNA extraction method to obtain cfDNA from plasma from whole blood. Extracted cfDNA underwent whole-genome shotgun library construction and hybridization- based capture of 324 cancer-related genes.

In this work, the genetic aberrations were ranked and rated according to the ESMO Scale for Clinical Actionability of molecular Targets (ESCAT) to objectify their value as clinical targets based on the available strength of evidence (15, 16).

### Immunohistochemistry

Immunohistochemistry was performed using 2-μm-thin tissue sections read by a Ventana Benchmark Ultra Stainer (Ventana, Tucson, Arizona, USA). The following antibody was applied: programmed death-ligand 1 (PD-L1) (clone E1L3N; Cell Signaling Technology).

The immunohistochemical staining intensity for *HER2* was scored from 0 to 3+ (0 = negative, 1+ = negative, 2+ = positive, 3+ = positive) according to the scoring guidelines of the Dako HercepTest™ (Agilent Technologies, Vienna, Austria). In the case of *HER2* 2+, further testing with *HER2 in situ* hybridization was performed to verify the *HER2* gene amplification.

For PD-L1, the tumor proportion score was calculated, which is the percentage of viable malignant cells with membrane staining.

The presence of MSI was assessed using the MSI Analysis System Version 1.1 (Promega Corporation, Madison, Wisconsin, USA).

### Descriptive statistics

For data description, we used measures of central tendency, including the mean and median. Furthermore, we used the method of frequency distribution to delineate the characteristics of the BTC patients.

## Results

In this tricentric analysis of the main Austrian centers Medical University of Vienna, University Hospital St. Poelten, and University Hospital Krems, the molecular profiles of 92 patients with metastatic BTC between June 2013 and January 2022 were evaluated. All patients were Caucasian, and the cohort comprised 52 men and 40 women.

The most common BTC subentity was iCCA, which was diagnosed in 59 patients (64.1%), followed by extrahepatic (distal and perihilar) CCA (n=23; 24.9%), gallbladder carcinoma (n=9; 9.8%) and ampullary carcinoma (n=1; 1.1%). The histological subtype in all patients was adenocarcinoma. The median age at first diagnosis was 63.3 years, ranging from 34.9 to 86.3 years. Twenty-four patients had a relapsed BTC. All the patients had distant metastases, including 60 patients with liver metastases, 19 patients with osseous metastases, 21 patients with peritoneal carcinomatosis, and four patients with pleural carcinomatosis. Metastases to the spleen, brain, and ovaries were reported in one patient each (**Table 1**).

**Table 1:** Patient characteristics (n=92).

Patient Characteristics	Number
Median age at fist diagnosis (years)	63.3
Median lines of treatment	2
Men	52 (56.5%)
Women	40 (43.5%)
Caucasian	92 (100%)
Metastatic disease	92 (100%)
Intrahepatic cholangiocarcinoma	59 (64.1%)
Perihilar cholangiocarcinoma	11 (11.9%)
Distal cholangiocarcinoma	12 (13.0%)
Gallbladder carcinoma	9 (9.8%)
Ampullary carcinoma	1 (1.1%)
Molecular profiling failed due to insufficient material	16 (17.4%)
Relapsed	24 (26.1%)

In total, we identified 205 molecular aberrations, including 198 mutations affecting 89 different genes in 61 patients. The predominant mutations were in *KRAS* (n=17; 22.4%), *TP53* (n=17; 22.4%), *PIK3CA* (n=7; 9.2%), *FGFR2* (n=7; 9.2%), *DNMT3A* (n=7; 9.2%), *IDH1* (n=7; 9.2%), *IDH2* (n=6; 7.9%), *CDKN2A* (n=6; 7.9%), *BAP1* (n=4; 5.3%), *NF1* (n=4; 5.3%), and *NF2* (n=4; 5.3%). These mutations accounted for 43.7% of all the mutations. *BRAF* mutations were observed in four patients, including one *BRAF V600E* mutation in a patient who was subsequently enrolled in the ROAR phase 2 trial (17).

None of the patients harbored the *KRAS* G12C mutation. MSI-H status and *FGFR2* fusion genes (*FGFR2::OFD1* and *FGFR2::DDX21*) were each observed in two different patients. Both patients with MSI-H status also had tumor mutational burden-high (TMB-H). All *IDH1* and *IDH2* mutations and both *FGFR2* fusions genes were found in iCCA patients. *HER2* positivity was reported in three patients, of whom two patients had gallbladder carcinoma. The aberrations were categorized according to the ESCAT classification (see **Table 2** for the complete list). Four patients underwent molecular analysis via liquid biopsy.

**Table 2:** Molecular aberrations and the applied targeted therapies.

ESCAT tier	Aberration	Patients	Prevalence among patients with successful molecular profiling	Targeted therapy applied	Name of targeted therapy	Response
X	*KRAS*	17	22.4%	0		
X	*TP53*	17	22.4%	0		
IIIA	*PIK3CA*	7	9.2%	0		
IVA	*FGFR2*	7	9.2%	0		
X	*DNMT3A*	7	9.2%	0		
IA	*IDH1*	7	9.2%	1	Ivosidenib	PD
IVA	*IDH2*	6	7.9%	0		
IVA	*CDKN2A*	6	7.9%	0		
X	*BAP1*	4	5.3%	0		
X	*NF1*	4	5.3%	0		
X	*NF2*	4	5.3%	0		
IVA	*ARID1A*	3	3,9%	0		
X	*BRAF* non *V600E*	3	3.9%	0		
X	*NOTCH2*	3	3.9%	0		
IIIA	*ERBB2*	3	3.9%	0		
IVA	*ATM*	3	3.9%	0		
IVA	*ATR*	3	3.9%	0		
IVA	*POLE*	3	3.9%	0		
IC	*HER2* positivity	3	3.9%	3	Trastuzumab emtansine Trastuzumab + pertuzumab Trastuzumab	Trastuzumab + pertuzumab resulted in progressive disease. The two other patients died prior to restaging.
X	*NRAS*	2	2.6%	0		
X	*FBXW7*	2	2.6%	0		
X	*SRC*	2	2.6%	0		
X	*SMAD4*	2	2.6%	0		
X	*RAD51B*	2	2.6%	0		
X	*NTRK1*	2	2.6%	0		
X	*E2F3*	2	2.6%	0		
IIIA	*BRCA1*	2	2.6%	0		
IIIA	*BRCA2*	2	2.6%	1	Olaparib	Progressive disease
X	*ATRX*	2	2.6%	0		
X	*FANCD2*	2	2.6%	0		
X	*FANCI*	2	2.6%	0		
X	*CREBBP*	2	2.6%	0		
IVA	*MET*	2	2.6%	0		
X	*PREX2*	2	2.6%	0		
X	*JAK1*	2	2.6%	0		
X	*SETD2*	2	2.6%	0		
X	*CCND1*	2	2.6%	0		
IB	*FGFR2* gene fusions	2	2.6%	2	Pemigatinib Regorafenib	Both patients achieved stable disease.
IC	MSI-H	2	2.6%	2	Pembrolizumab	Both patients achieved a complete response.
X	*PIK3R1*	2	2.6%	0		
X	*PRKDC*	2	2.6%	0		
X	*MDM2*	1	1.3%	0		
X	*MDM4*	1	1.3%	0		
X	*PIK3CB*	1	1.3%	0		
X	*PIK3C2G*	1	1.3%	0		
IVA	*FGFR1*	1	1.3%	0		
X	*FGF2*	1	1.3%	0		
X	*FGF3*	1	1.3%	0		
X	*FGF19*	1	1.3%	0		
IB	*BRAF V600E*	1	1.3%	1	Dabrafenib + trametinib	Partial response in the ROAR trial
IVA	*CDK2*	1	1.3%	0		
IVA	*CDK4*	1	1.3%	0		
X	*CDK12*	1	1.3%	0		
X	*SMARCA4*	1	1.3%	0		
X	*NTRK3*	1	1.3%	0		
X	*AR*	1	1.3%	0		
X	*NOTCH3*	1	1.3%	0		
X	*NOTCH4*	1	1.3%	0		
X	*ERBB3*	1	1.3%	0		
X	*PDGFRB*	1	1.3%	0		
X	*FANCA*	1	1.3%	0		
X	*MYC*	1	1.3%	0		
X	*MYCL*	1	1.3%	0		
X	*MYCN*	1	1.3%	0		
X	*CRKL*	1	1.3%	0		
X	*PTCH1*	1	1.3%	0		
X	*JAK2*	1	1.3%	0		
X	*RNF43*	1	1.3%	0		
X	*CIC*	1	1.3%	0		
X	*NSD1*	1	1.3%	0		
X	*NFE2L2*	1	1.3%	0		
X	*DNMT3B*	1	1.3%	0		
X	*TSC1*	1	1.3%	0		
X	*STK11*	1	1.3%	0		
X	*INHBA*	1	1.3%	0		
X	*NKX2*	1	1.3%	0		
X	*STAG2*	1	1.3%	0		
X	*ABL1*	1	1.3%	0		
X	*RAD50*	1	1.3%	0		
X	*TFGBR2*	1	1.3%	0		
X	*RASA1*	1	1.3%	0		
X	*TET1*	1	1.3%	0		
X	*SLX4*	1	1.3%	0		
X	*CTNNB1*	1	1.3%	0		
X	*CSF1R*	1	1.3%	0		
X	*STAT5A*	1	1.3%	0		
X	*CASP8*	1	1.3%	0		
X	*SOX17*	1	1.3%	0		
X	*LATS1*	1	1.3%	0		
X	*ROS1*	1	1.3%	0		
X	*BCOR*	1	1.3%	0		
X	*MLL2*	1	1.3%	0		

No mutations were detected in 15 patients, including 9 patients with iCCA, 6 patients with pCCA, 1 patient with gallbladder carcinoma and 1 patient with ampullary carcinoma.

2Molecular profiling failed in 16 (17.4%) patients due to lacking or insufficient material. Based on the current ESCAT classification, 19 patients had a targetable molecular aberration with at least ESCAT III tier, representing 25% of all patients (n=76) with a successful molecular analysis (**Table 2**).

In more than three-fourths (n=69) of the patients, molecular profiling was performed after failure of standard treatments. The median turnaround time in these patients from the decision to perform molecular profiling to the initiation of the targeted therapy was 49 days.

Seventy-six (82.3%) patients received a platinum-based chemotherapy in the first line. The median applied lines of treatment were two (ranging from one to five).

Eventually, 10 patients received targeted therapy based on the individual molecular profile. The patient harboring the *BRAF V600E* mutation achieved partial response under dabrafenib plus trametinib in the previously published phase 2 ROAR basket trial (17).

Both patients with MSI-H status treated with pembrolizumab achieved complete response. Pembrolizumab treatment is still ongoing in one patient at the time of publication of this report; in the other patient, pembrolizumab treatment was terminated, and he is receiving oncological aftercare.

**Figure 1:**
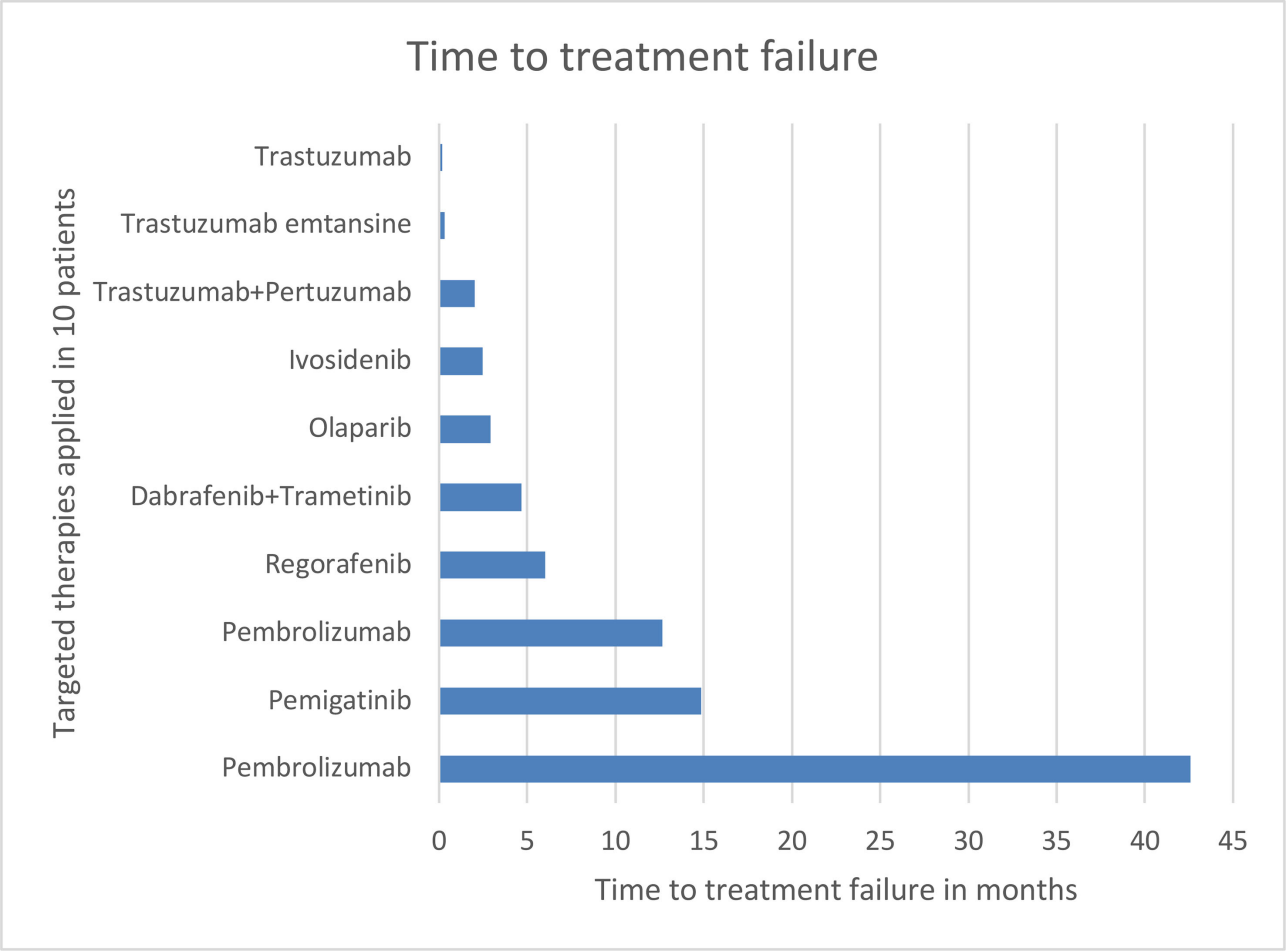
Time to treatment failure in 10 patients treated with a targeted therapy.

One patient with *FGFR2::OFD1* gene fusion was treated with pemigatinib and achieved stable disease. The other patient with *FGFR2::DDX21* continues to receive first line treatment. The patient with *FGFR2* mutation received regorafenib and achieved stable disease. None of the patients with *HER2* amplification with a *HER2* score of 3+ who were treated with an anti-*HER2* directed therapy achieved a clinical benefit. Olaparib was administered to a patient with *BRCA2* mutation; however, he did not respond. Ivosidenib was used to treat an iCCA patient with an *IDH1* mutation; however, he experienced progressive disease. See **Figure 1** for time to treatment failure.

## Discussion

In this retrospective, tricentric, real-world analysis, we examined the molecular profiles of 92 patients with BTC. Our work demonstrated that molecular profiling is feasible and implementable in routine clinical practice at university hospitals. We found 198 mutations affecting 89 different genes, which reflects the molecular heterogeneity of BTC. This finding is consistent with the well-described extreme and complex intratumoral heterogeneity of BTC that occurs within the same tumor tissue; vascularization, proliferation, and subclones are all known to be highly variable. The pattern of genetic and epigenetic aberrations varies both spatially and temporally. Tumor biology at metastatic sites is different from the primary site and varies in a relapse (4, 9, 18–23). The mutations identified in this analysis are in line with previous reports. All *IDH1* mutations and *FGFR2* fusions were found in iCCA. A growing body of evidence reports that the genomic landscape of BTC differs with the location of the carcinoma. Intrahepatic CCA is frequently associated with genomic aberrations in *IDH1*, *IDH2*, and *BAP1*, while perihilar and distal CCA are frequently associated with mutations in *KRAS*, *TP53*, and *SMAD4* (24–29). Mutations in *KRAS, TP53, APC, SMAD4* are frequently seen in ampullary carcinoma (30). *HER2* amplifications are mainly found gallbladder carcinoma (31). In our multicentric cohort, three patients displayed HER2 positivity, of whom two patients had gallbladder carcinoma. Based on the current ESCAT classification, 19 patients had a targetable molecular aberration with at least ESCAT III tier, which represents 25% of all patients with a successful molecular analysis. This result underscores that BTC is a target-rich disease (24).

Ten patients received targeted therapy. The drugs were carefully selected for individualized treatment, with consideration of the patient’s molecular information, clinical and treatment history, performance status, comorbidities, and concomitant therapies.

Both patients with MSI-H status were treated with pembrolizumab and achieved complete response, demonstrating a deep and durable clinical benefit. MSI-H is a predictive marker for immunotherapy and is found in less than 5% of BTC patients (32). The multicohort phase 2 trial KEYNOTE-158 included 22 pretreated BTC patients with MSI-H who were treated with pembrolizumab (33). An ORR of 40.9% was achieved. The mPFS and mOS were 4.2 and 24.3 months, respectively. Median DOR was not reached (34). Based on these results, pembrolizumab received the first tumor tissue-agnostic approval from the FDA for the treatment of MSI-H-positive solid tumors in 2017 (35). Recently, the EMA granted approval for this treatment for MSI-H-positive BTC patients who have disease progression during or following at least one prior therapy.

The promising results emphasize the importance of determining the status of microsatellite stability alongside genetic testing.

Although our analysis showed that molecular profiling is implementable in routine clinical practice, only five patients derived a clinical benefit through this approach. There are several reasons for this modest outcome. One reason may be the relatively long median turnaround time of nearly 50 days from the decision to perform molecular profiling after failure of all standard therapies to the initiation of the targeted therapy in patients for whom there was no effective therapy for a highly aggressive cancer. This might explain why two patients who received anti-*HER2* therapy died prior to restaging; the targeted therapy was initiated too late to exhibit its full antitumoral potential.

Upfront testing would be reasonable to bridge the turnaround time while the patient is under treatment. In addition, liquid biopsy may be a viable option as it supersedes the need for conventional biopsy, which prolongs the turnaround time due to the need to organize inpatient admission and is associated with intervention-related complications. Therefore, liquid biopsy may help to reduce turnaround time, monitor the disease, and assess the response to therapy (36). Another reason for the modest response may be the complexity of tumor biology, reflected in part by the high degree of heterogeneity of BTC (3, 37, 38).

The molecular profiles of the BTC patients were collected between 2013 and 2022. For a large part of this period, the clinical actionability of the identified molecular lesions were not ranked or standardized. It was not until 2018 that ESCAT tiers to rank and classify the targetable aberrations. Certain molecular alterations were initially not targetable and have only recently become druggable. A prime example is *IDH1* mutation. The phase 3 ClarIDHy trial, published in 2020, demonstrated the clinical benefits of ivosidenib in *IDH1*-mutant, chemotherapy-refractory BTC (27, 39). Seven patients in our analysis were *IDH1* mutants; however, only one patient received molecular-guided therapy with ivosidenib since this mutation was identified at a time when ivosidenib was accessible through a compassionate use program. This example demonstrates the advances in precision and molecular oncology in the field of BTC (40).

According to the current ESCAT classification, 19 patients had a targetable molecular aberration with at least ESCAT III tier, representing 25% of all patients in our cohort with a successful molecular analysis. Thus, molecular profiling strongly informs the clinical decision finding, particularly after the failure of the-first line therapy. In future, more and more BTC patients will benefit from molecular profiling and precision oncology due to three main reasons:

Firstly, biomarkers such as *IDH1* and *FGFR2* fusions are currently investigated in clinical trials in first-line settings which means that - in case that the trials meet the endpoint – the patients will receive front-up a targeted therapy (41–43). The second reason is that new predictive biomarkers are tested in different trials, including biomarkers for predicting the effectiveness of immunotherapies, such as *ATM, ATR, BRCA1, BRCA2, FANCA, and POLE* (44, 45). Last, but not least, new emergent therapies will likely be a gamechanger in the BTC management, particularly *KRAS* inhibitors (46, 47).

Another reason for the modest response in our study is the remarkable percentage (17.4%) of failure of molecular profiling due to insufficient material, which is comparable with the percentage reported by Lamarca et al. (48).

These explanations highlight the importance of performing upfront testing in all BTC patients, as in case of failure, early testing would allow enough time to re-biopsy the patient to collect sufficient material for repeat testing. Further, upfront testing now has a therapeutic consequence as it impacts therapy sequencing after failure of the first line therapy (48).

This study has an important limitation: it was a non-randomized, retrospective analysis of patients without an adequate control group. However, this study demonstrated the potential and challenges of precision oncology in a real-world setting for BTC management.

Molecular profiling and molecular oncology are integral elements of the modern therapeutic management of BTC patients and should be implemented as upfront testing in routine clinical practice.

## Data availability statement

The datasets presented in this study can be found in online repositories. The names of the repository/repositories and accession number(s) can be found in the article/supplementary material.

## Ethics statement

The studies involving human participants were reviewed and approved by Kommission für scientific integrity und Ethik der Karl Landsteiner Privatuniversität. Written informed consent for participation was not required for this study in accordance with the national legislation and the institutional requirements.

## Author contributions

Contributions: (I) Conception and Design: HT, GP (II) Administrative support: All authors (III) Provision of study materials or patients: All authors (IV) Collection and assembly of data: HT (V) Data analysis and interpretation: All authors (VI) Manuscript writing: All authors; All authors contributed to the article and approved the submitted version.
